# HLA frequency and prognosis in lung cancer.

**DOI:** 10.1038/bjc.1981.90

**Published:** 1981-05

**Authors:** C. H. Ford, C. E. Newman, P. Mackintosh

## Abstract

In 100 patients with lung cancer we have found no significant abnormality in overall HLA antigen frequency when compared to a control sample of 151 random health individuals from the same region, though there was a high relative risk of being HLA-BW22-positive and having lung cancer. There was an increased frequency of HLA-B5 in small-(oat-)cell anaplastic carcinomas (P less than 0.05); HLA-B15 in anaplastic tumours (P less than 0.05); HLA-B40 in Stage III patients (P = 0.05) and a decreased frequency of HLA-B12 in adenocarcinomas (P less than 0.05). In 86 patients followed up for 2 1/2-5 3/4 years after surgery we have been unable to confirm the significant association of HLA-AW19 and/or HLA-B5 with good prognosis as reported by others. The most striking observation was that the frequency of HLA-BW22 was significantly higher in patients alive at least 2 1/2 years after surgery when compared to the control groups (P less than 0.05) and 83% of patients HLA-BW22-positive are alive compared to only 52.5% of lung cancer patients lacking this antigen. However, all the P values become nonsignificant when multiplied by the number of antigens studied, and these observations need further investigation in a large, prospective study.


*Br. J. Cancer* (1981) **43**, 610

### HLA FREQUENCY AND PROGNOSIS IN LUNG CANCER

C. H. J. FORD*, C. E. NEWMAN* AND P. MACKINTOSH†

*From the *Surgical Immunology Unit, Clinical Oncology, University of Birmingham, Queen Elizabeth Hospital, and †Tissue Typing Laboratory, Blood Transfusion Service, Vincent Drive, Edgbaston, Birmingham*

Received 8 December 1980 Accepted 26 January 1981

**Summary.—In 100 patients with lung cancer we have found no significant abnormality in overall HLA antigen frequency when compared to a control sample of 151 random healthy individuals from the same region, though there was a high relative risk of being HLA-BW22-positive and having lung cancer. There was an increased frequency of HLA-B5 in small-(oat-)cell anaplastic carcinomas ($P<0{\cdot}05$); HLA-B15 in anaplastic tumours ($P<0{\cdot}05$); HLA-B40 in Stage III patients ($P=0{\cdot}05$) and a decreased frequency of HLA-B12 in adenocarcinomas ($P<0{\cdot}05$). In 86 patients followed up for $2\frac{1}{2}$–$5\frac{3}{4}$ years after surgery we have been unable to confirm the significant association of HLA-AW19 and/or HLA-B5 with good prognosis as reported by others. The most striking observation was that the frequency of HLA-BW22 was significantly higher in patients alive at least $2\frac{1}{2}$ years after surgery when compared to the control group ($P<0{\cdot}05$) and 83% of patients HLA-BW22-positive are alive compared to only 52·5% of lung cancer patients lacking this antigen. However, all the *P* values become non-significant when multiplied by the number of antigens studied, and these observations need further investigation in a large, prospective study.**

When the risk data from several studies of HLA association with malignancy are combined, there is an indication of an association of HLA-A1 with Hodgkin's disease, HLA-A2 with acute lymphocytic leukaemia, and Sin 2 (an HLA-B antigen) and Sin 2a (an HLA-D antigen) with nasopharyngeal carcinoma in Singapore Chinese (reviewed by Rogentine, 1979). In most of the studies in lung cancer, however, no significant differences in HLA antigen frequency have been found at diagnosis, though there have been several reports of HLA association with prognosis.

In a retrospective study of 14 surgically cured patients it was reported that HLA-AW19 and HLA-B5 were associated with a better prognosis (Dellon *et al.*, 1975). In patients who were prospectively typed they also found a clear association of these two antigens with prognosis in patients with squamous or adenocarcinomas. This was confirmed in a 2-year follow-up study, when patients with either antigen had a 57% (12/21) chance of being alive and disease free 2 years after diagnosis, compared to only a 13% chance (6/48) if they lacked these antigens (Rogentine *et al.*, 1977). This observation has been complemented by a report of a retrospective study of 20 patients with non-oat-cell bronchogenic carcinoma of the lung, alive at least 1 year after diagnosis, in which 50% of the survivors were found to have one or other of these antigens (Weiss *et al.*, 1980).

However, in another report 20 retrospectively typed patients who had survived more than a year after surgery were found to have an increased frequency of HLA-B8 (Sengar *et al.*, 1977). These

Correspondence: Dr C. H. J. Ford, Surgical Immunology Unit, Clinical Oncology, Queen Elizabeth Hospital, Edgbaston, Birmingham B15 2TH.

authors also reported a decreased frequency of HLA-A2 in the 37 patients they studied. More recently a prospective and retrospective study was reported in which the frequency of HLA-B12 was found to be raised in all patients, and HLA-A29 was found to be raised in patients who had died, whereas the frequency was normal in 5-year survivors (Tongio *et al.*, 1980).

The aim of this study was to investigate the frequency of HLA antigens in people with bronchogenic carcinoma and to determine whether the reported associations with prognosis applied to the lung-cancer patients in this region.

### MATERIALS AND METHODS

*Patients and controls.*—Eighty-six patients with lung cancer of various histological types, who were eligible for entry into a controlled trial of passive immunochemotherapy after resection of their cancer (Newman *et al.*, 1977), and 14 patients with small-cell anaplastic carcinoma of the lung who entered a pilot study of chemotherapy and radiotherapy, were investigated. Peripheral-blood lymphocytes were obtained before surgery or other therapeutic procedures and used for typing immediately, or frozen down in liquid $N_2$ and stored for typing at a later date. Details of the freezing, storage and thawing procedures have been published elsewhere (Ford *et al.*, 1979). In cases where pre-treatment blood samples were not available, lymphocytes were obtained from blood samples taken during outpatient follow-up. For the 86 patients in the immunochemotherapy study there is a minimum follow-up of $2\frac{1}{2}$ years and a maximum of $5\frac{3}{4}$ years after treatment. Forty-seven of these patients are still alive at least $2\frac{1}{2}$ years after surgery. A group of 151 random healthy individuals from this district were tested as a control group for this study.

*HLA typing.*—Antisera recognizing 24 HLA A and B specificities were used. For some of the antigens (*e.g.* AW19 and B12) antisera defining individual sub-specificities were used, but since not all of the patients were tested with these antisera only the main groups have been considered in the analysis. When there was any ambiguity in the typing result it was repeated. The standard NIH micro-lymphocytotoxic test was used (NIAID Manual of Tissue Typing Techniques, 1976).

*Analyses.*—HLA antigen frequencies were compared between groups by means of $\chi^2$ analysis using Yate's correction. Relative liability (Edwards, 1974) was also plotted.

### RESULTS

The percentage frequencies of HLA antigens in the 100 lung-cancer patients and 151 controls are shown in Table I, together with the frequency in the different histological types. For the 86 patients for whom there is at least a $2\frac{1}{2}$-year exposure to risk of recurrence after surgery the antigen frequency in those alive and dead has been calculated, as well as the frequency in Stage I and Stage III patients (there were only 6 Stage II patients).

Comparison of the overall lung-cancer group with the control group shows no significant difference in the frequency of the antigens studied, though the percentages differ. There were significant differences between the lung-cancer group and controls for: an increased frequency of HLA-B15 in anaplastic tumours ($P < 0{\cdot}05$); a decreased frequency of HLA-B12 in adenocarcinomas ($P < 0{\cdot}05$); an increased frequency of HLA-B5 in small-(oat-) cell anaplastic carcinomas ($P < 0{\cdot}05$) and an increased frequency of HLA-B40 in Stage III patients ($P = 0{\cdot}05$).

Although none of the antigens was associated with lung cancer, from Fig. 1 it can be seen that there is a higher relative risk of being HLA-BW22 positive and having lung cancer (plotting 3-fold above the median line). The low frequency of this antigen in the normal population (3/151) and the small number of cancer patients positive for HLA-BW22 (6/100) makes this observation non-significant. However, when the frequency of HLA-BW22 in the 47 patients alive at least $2\frac{1}{2}$ years after surgery is compared with the normal population, there is a significant increase in the frequency of the antigen in the survivors (plotting more than 4-fold above the median line ($P < 0{\cdot}05$) (Fig. 2).

In Table II HLA frequency and survival

TABLE I.—% *of HLA antigens in lung-cancer patients and controls*

| HLA antigens | Control (n=151) | All lung-cancer patients (n=100) | Histological type*: ADENO (n=17) | ANAPL (n=15) | SCA (n=23) | SCC (n=45) | Patients with a minimum follow-up of 2½ years: Total (n=86) | Alive† (n=47) | Dead‡ (n=39) | Stage I (n=49) | Stage III (n=31) |
|---|---|---|---|---|---|---|---|---|---|---|---|
| A1 | 38 | 31 | 35 | 33 | 39 | 24 | 30 | 28 | 33 | 24 | 35 |
| A2 | 54 | 50 | 35 | 40 | 43 | 62 | 52 | 53 | 51 | 51 | 58 |
| A3 | 23 | 24 | 29 | 27 | 22 | 22 | 23 | 23 | 23 | 20 | 29 |
| A9 | 15 | 22 | 29 | 13 | 26 | 20 | 22 | 26 | 18 | 24 | 16 |
| A10 | 7 | 9 | 18 | 13 | 13 | 2 | 7 | 11 | 3 | 8 | 6 |
| A11 | 12 | 11 | 0 | 27 | 17 | 7 | 9 | 11 | 8 | 8 | 6 |
| A28 | 7 | 2 | 12 | 0 | 0 | 0 | 2 | 2 | 3 | 4 | 0 |
| AW19 gp. | 17 | 19 | 24 | 20 | 17 | 18 | 17 | 13 | 26 | 22 | 10 |
| B5 | 9 | 12 | 6 | 7 | 26 | 9 | 9 | 9 | 10 | 6 | 13 |
| B7 | 27 | 25 | 35 | 20 | 17 | 27 | 27 | 28 | 26 | 27 | 32 |
| B8 | 26 | 20 | 24 | 13 | 22 | 20 | 20 | 15 | 26 | 24 | 16 |
| B12 | 40 | 29 | 12 | 27 | 35 | 33 | 28 | 32 | 23 | 29 | 29 |
| B13 | 2 | 3 | 0 | 0 | 4 | 4 | 2 | 2 | 3 | 2 | 3 |
| B14 | 10 | 6 | 12 | 7 | 0 | 7 | 7 | 9 | 5 | 8 | 3 |
| B15 | 11 | 14 | 6 | 33 | 9 | 13 | 14 | 17 | 10 | 12 | 16 |
| BW16 | 3 | 6 | 6 | 13 | 9 | 2 | 6 | 4 | 8 | 4 | 9 |
| B17 | 7 | 13 | 18 | 7 | 13 | 13 | 13 | 13 | 13 | 14 | 9 |
| B18 | 5 | 4 | 18 | 0 | 0 | 2 | 5 | 4 | 5 | 8 | 0 |
| BW21 | 4 | 6 | 12 | 0 | 4 | 7 | 6 | 4 | 8 | 4 | 9 |
| BW22 | 2 | 6 | 6 | 7 | 0 | 9 | 7 | 11 | 3 | 8 | 6 |
| B27 | 8 | 9 | 0 | 13 | 9 | 11 | 9 | 9 | 10 | 14 | 0 |
| BW35 | 13 | 8 | 12 | 13 | 9 | 7 | 8 | 4 | 13 | 4 | 13 |
| B37 | 2 | 2 | 0 | 0 | 0 | 4 | 2 | 2 | 3 | 0 | 6 |
| B40 | 9 | 14 | 24 | 13 | 17 | 9 | 13 | 6 | 21 | 6 | 23 |

* ADENO = adenocarcinomas; ANAPL = anaplastic carcinomas; SCA = small-(oat-)cell anaplastic carcinomas; SCC = squamous-cell carcinomas.

† 8 ADENO; 10 ANAPL; 3 SCA; 26 SCC.

‡ 9 ADENO; 5 ANAPL; 6 SCA; 19 SCC.

FIG. 1.—This computer diagram (Edwards, 1974) shows the relative liability to lung cancer for each antigen and the standard error of the estimate in 100 lung-cancer patients compared with 151 controls (the sizes of the squares are proportional to the numbers of individuals of each phenotype).

are presented for antigens that others have claimed to be associated with prognosis, as well as HLA-BW22, using a similar format to Rogentine *et al.* (1977). In the 86 patients for whom we have long-term follow-up, HLA-AW19, HLA-B5 or a combination of the two are not associated with a better prognosis than patients without these antigens. When only squamous and adeno-carcinomas are considered, again there is no association with a better prognosis. In fact, in both groups

FIG. 2.—As Fig. 1, but comparing the survival for at least 2½ years from surgery of 47 lung-cancer patients with 151 controls.

TABLE II.—*HLA and survival*

| Patient group | Survival category | Antigens: AW19 | B5 | AW19 and/or B5 | Neither |
|---|---|---|---|---|---|
| All patients (n = 86) | Alive | (n = 16) | (n = 8) | (n = 22) | (n = 64) |
| | | 37·5% (6)* | 50% (4) | 45% (10) | 58% (37) |
| | Dead | 62·5% (10) | 50% (4) | 55% (12) | 42% (27) |
| Squamous and adeno-carcinomas (n = 62) | Alive† | (n = 12) | (n = 5) | (n = 15) | (n = 47) |
| | | 41·7% (5) | 60% (3) | 53·3% (8) | 55·3% (26) |
| | Dead | 58·3% (7) | 40% (2) | 46·7% (7) | 44·7% (21) |
| | | B8 | Not B8 | BW22 | Not BW22 |
| All patients (n = 86) | Alive | (n = 17) | (n = 69) | (n = 6) | (n = 80) |
| | | 41% (7) | 58% (40) | 83% (5) | 52·5% (42) |
| | Dead | 59% (10) | 42% (29) | 17% (1) | 47·5% (38) |

* Numbers in parantheses.
† Two are alive with recurrence, but neither are AW19- or B5-positive.

HLA-AW19 is associated with a worse prognosis than that in patients lacking this antigen (non-significant). Similarly, in all 86 patients, HLA-B8 does not appear to be associated with a better prognosis. Patients positive for HLA-BW22 have an 83% chance of being alive at least 2½ years after surgery, compared to a 52·5% chance for patients with other antigens. This difference is not significant, probably because of the small numbers of HLA-BW22-positive individuals.

### DISCUSSION

In this study we have not found any significant deviation in antigen frequency for lung-cancer patients when compared to a control group from the same region. There is an indication of an increase of HLA-B15 in anaplastic tumours; a decrease of HLA-B12 in adenocarcinomas; an increase of HLA-B5 in small-cell anaplastic carcinomas and an increase of HLA-B40 in Stage III patients. However, if each *P* value for these significant differences is multiplied by the number of antigens looked at, they all become non-significant. This, together with the relatively small numbers in the subgroups, makes us very cautious in putting too much emphasis on the apparent associations.

An interesting observation was the increased frequency (non-significant) of HLA-BW22 in the lung cancer group and in particular the significant association of this antigen with patients alive at least

$2\frac{1}{2}$ years after surgery. Again, if the $P$ value is multiplied by the number of antigens looked for, it becomes non-significant. Also, because the frequency of HLA-BW22 is low in the normal population (3/151) and the number of cancer patients positive for this antigen is also small (6/100; or 5/47 in survivors) conclusions regarding the significance of the association with a better prognosis must be tentative.

A second finding was that in patients with a minimum follow-up of $2\frac{1}{2}$ years and a maximum of $5\frac{3}{4}$ years from surgery we have been unable to confirm the significant association of HLA-AW19 and/or HLA-B5 with a good prognosis, as reported by the NIH group for squamous and adeno-carcinomas of the lung (Dellon *et al.*, 1975; Rogentine *et al.*, 1977) and supported by Weiss *et al.* (1980). Similarly, we found no association of HLA-B8 with better prognosis in this group of patients, although Sengar *et al.* (1977) have reported such an association. The best association was seen for HLA-BW22, when 83% of antigen-positive individuals were alive at least $2\frac{1}{2}$ years after surgery compared with 52·5% of patients positive for antigens other than HLA-BW22, though this was not significant.

Whether this lack of correlation with other studies is due to differences in the population studied, the size of the groups, or the length of follow-up is unclear. Probably all three factors influence the results. The patient population studied for prognosis was heterogeneous with respect to tumour histology. As might be expected, there were more SCA patients in the dead group than in those alive, but overall the number of anaplastic, squamous and adeno-carcinomas were similar in those alive and dead. Four of the alive BW22-positive patients were Stage I. However, 46% of dead patients were Stage I and none of these were BW22-positive, so association with a particular stage appears unlikely.

Although the study was not performed retrospectively it is not entirely prospective, in that not all the lymphocyte samples were obtained before treatment. We feel these data indicate the need for larger, prospective studies to resolve some of these questions, especially with regard to HLA-BW22. Any association of an antigen with prognosis may aid considerably in our ability to tailor the treatment to individual patients, as well as giving a clue to genetic factors which may be involved in susceptibility or resistance to lung cancer. The apparent associations for other antigens with histology and stage would also be clarified by such studies.

We are grateful for financial support from the Endowment Fund of the Central Birmingham Health District, the West Midlands Regional Health Authority, The Chest Heart and Stroke Association and the Cancer Research Action Group.

We thank Miss S. Jobson and Mrs J. Fleming for their excellent assistance with this project and Miss Margot Morris for typing the manuscript.

### REFERENCES

Dellon, A. L., Rogentine, G. N., Jr & Chretien, P. B. (1975) Prolonged survival in bronchogenic carcinoma associated with HL-A antigens W-19 and HL-A5: A preliminary report. *J. Natl Cancer Inst.*, **54**, 1283.

Edwards, J. H. (1974) HL-A and disease: The detection of associations. *J. Immunogenet.*, **1**, 249.

Ford, C. H. J., Newman, C. E. & Carter, A. B. (1979) The effect of cryopreservation of lymphocytes on E rosetting ability: A study in lung cancer patients and controls. *J. Immunol. Methods*, **26**, 113.

Newman, C. E., Ford, C. H. J., Davies, D. A. L. & O'Neill, G. J. (1977) Antibody–drug synergism (ADS): An assessment of specific passive immunotherapy in bronchial carcinoma. *Lancet*, ii, 163.

NIAID (1976) NIH lymphocyte microcytotoxicity technique. In *Manual of Tissue Typing Techniques*. DHEW Publication No. **78-545**, 22.

Rogentine, G. N., Jr, Dellon, A. L. & Chretien, P. B. (1977) Prolonged disease free survival in bronchogenic carcinoma associated with HLA-AW19 and HLA-B5. A two-year prospective study. *Cancer*, **39**, 2345.

Rogentine, G. N., Jr (1979) HLA and blood group antigens. In *Immunodiagnosis of Cancer, Part 1.* Eds Herberman & McIntire. New York: Marcel Dekker. p. 669.

Sengar, D. P. S., McLeish, W. A., Stewart, T. H. M. & Harris, J. E. (1977) HLA antigens in bronchogenic carcinoma. *Oncology*, **34**, 143.

Tongio, M. M., Kerschen, C., Roeslin, P. G., Warter, A. & Mayer, S. (1980) HLA antigens and primary bronchial carcinoma. *4th Int. Cong. Immunol.* Abs 8.5.66.

Weiss, G. B., Nawrocki, L. B. & Daniels, J. C. (1980) HLA type and survival in lung cancer. *Cancer*, **46**, 38.


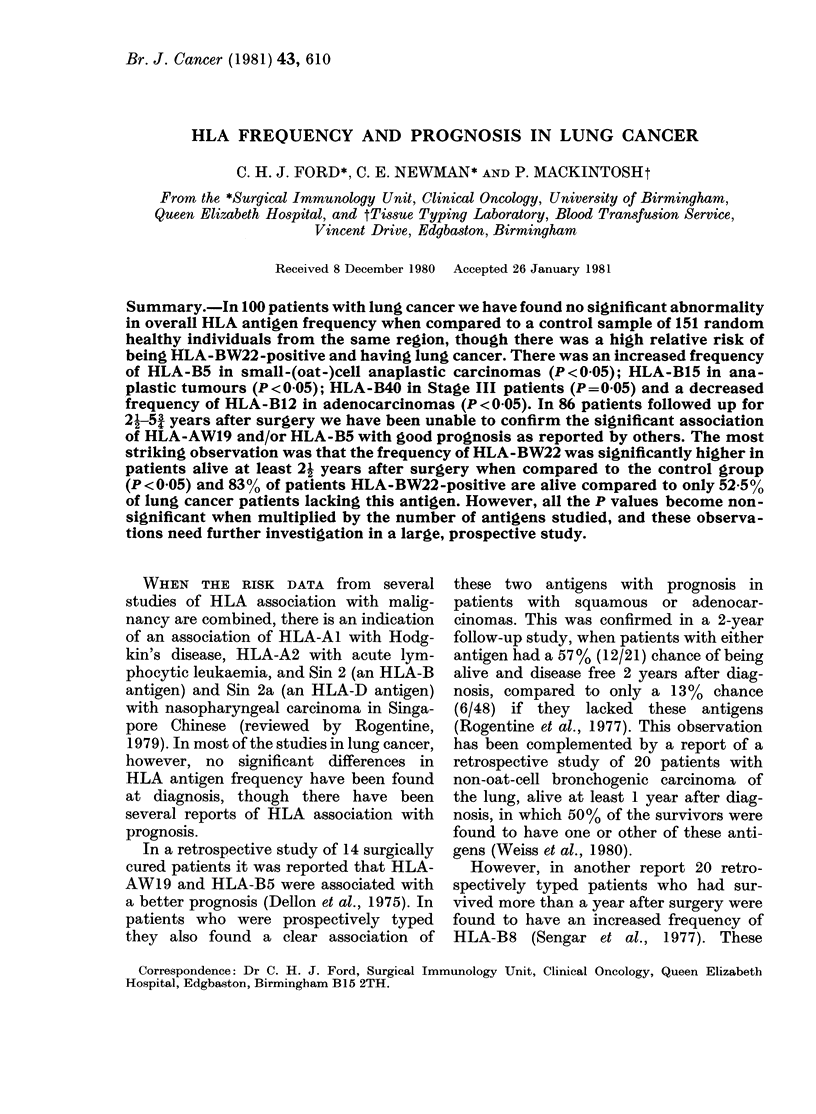

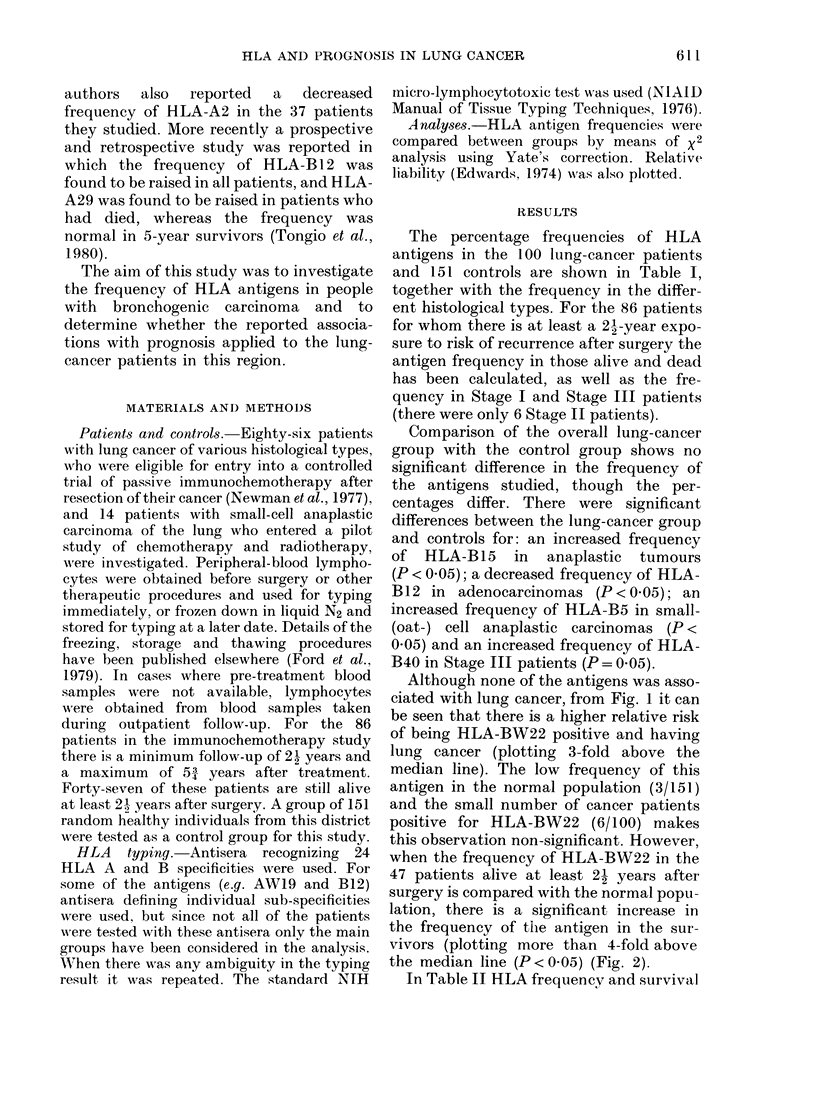

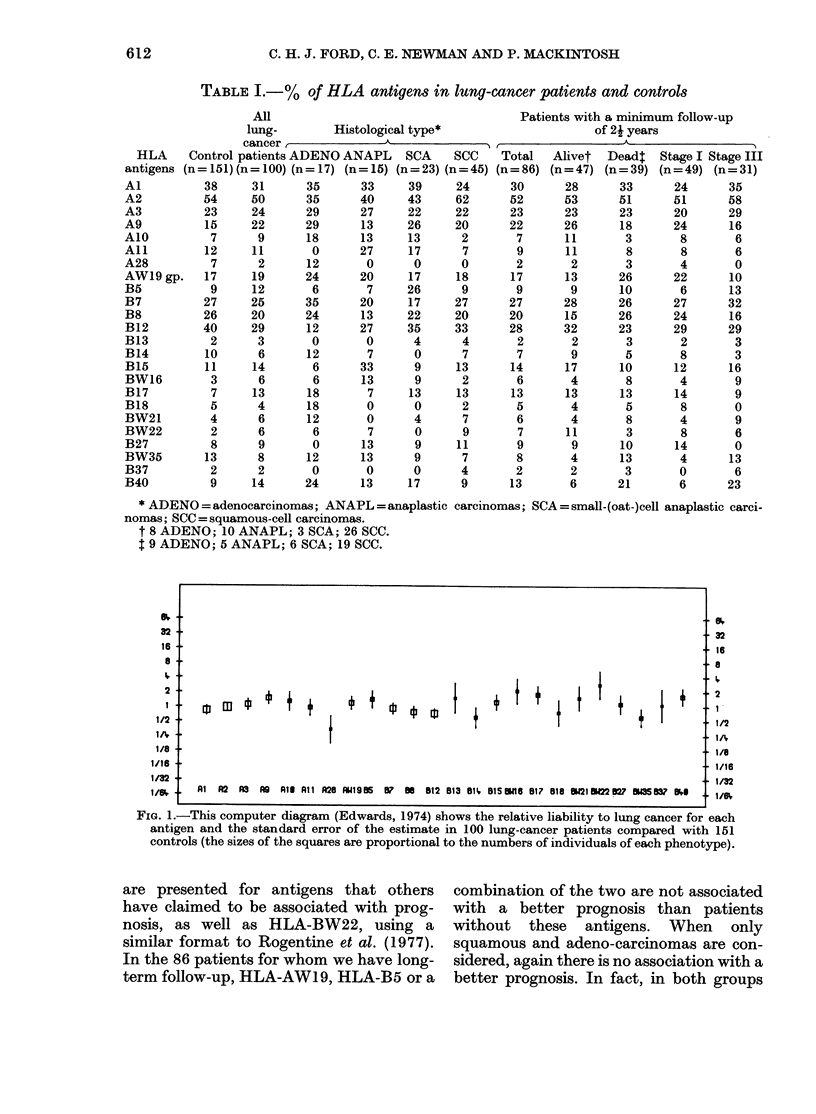

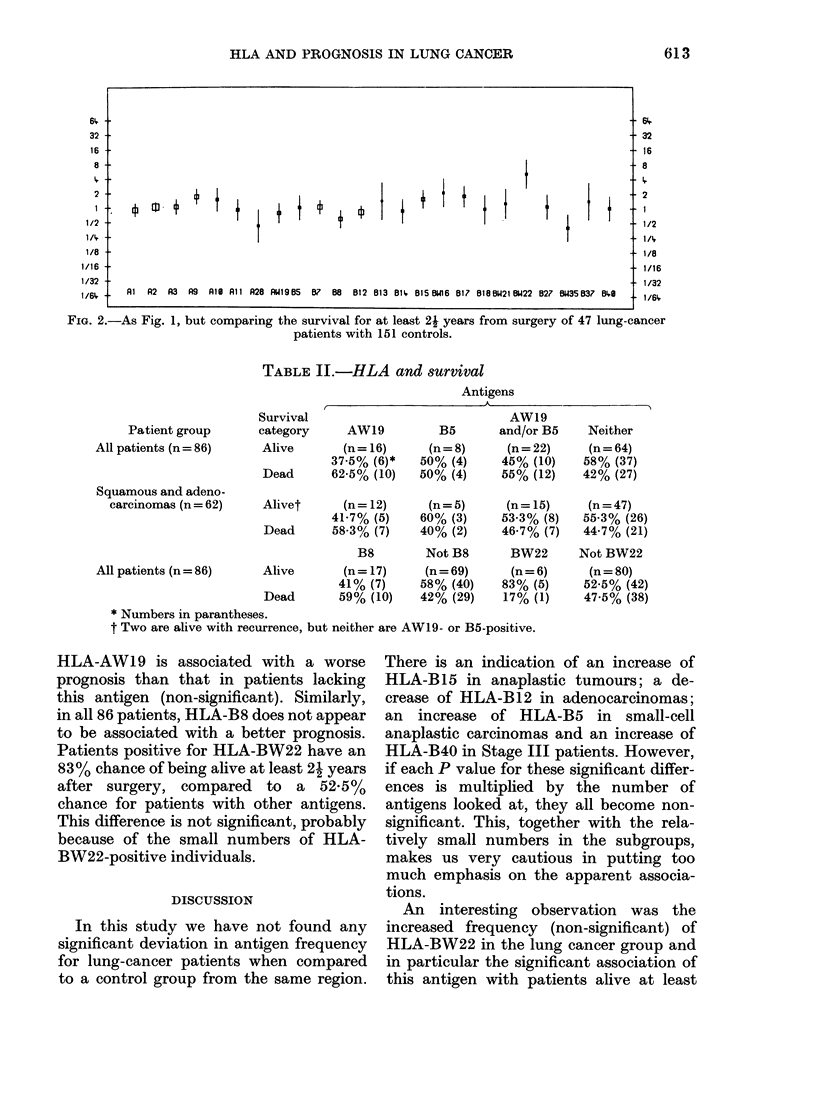

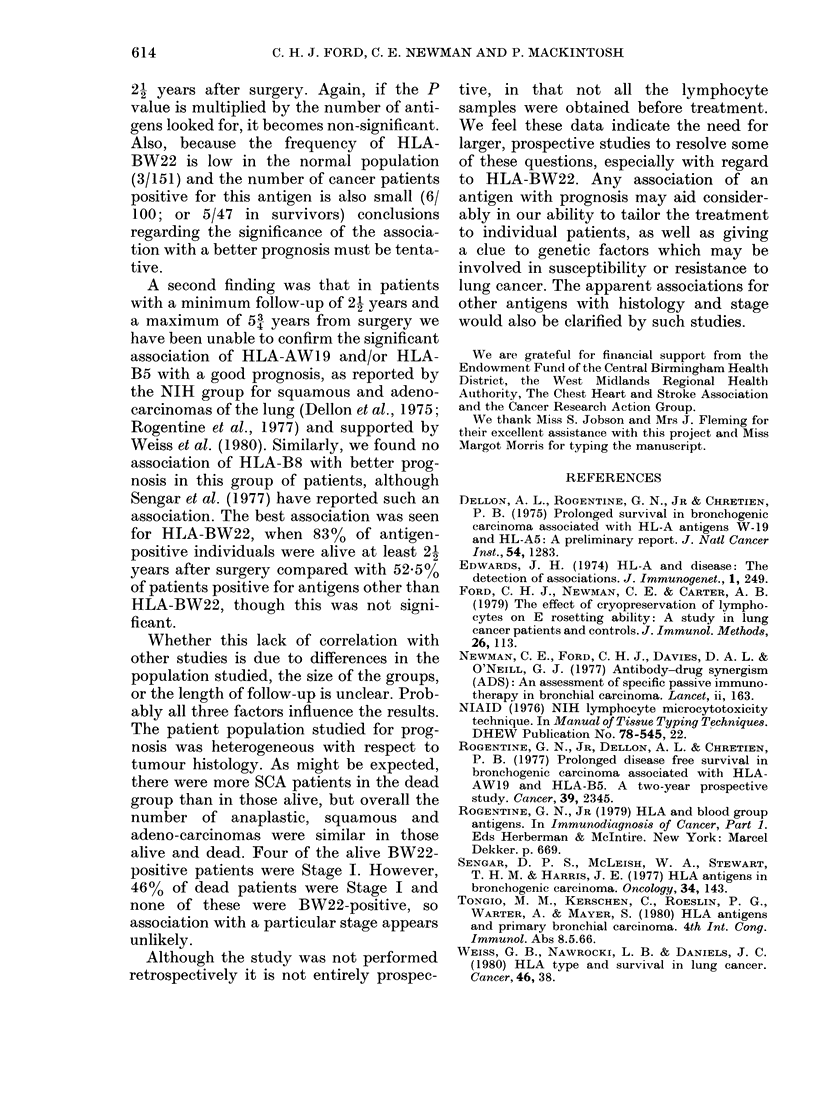


## References

[OCR_00486] Dellon A. L., Rogentine G. N., chretien P. B. (1975). Prolonged survival in bronchogenic carcinoma associated with HL-A antigens W-19 and HL-A5: a preliminary report.. J Natl Cancer Inst.

[OCR_00496] Ford C. H., Newman C. E., Carter A. B. (1979). The effect of cryopreservation of lymphocytes on E rosetting ability: a study in lung cancer patients and controls.. J Immunol Methods.

[OCR_00503] Newman C. E., Ford C. H. (1977). Antibody-drug synergism: An assessment of specific passive immunotherapy in bronchial carcinoma.. Lancet.

[OCR_00514] Rogentine C. N., Dellon A. L., Chretien P. B. (1977). Prolonged disease-free survival in bronchogenic carcinoma associated with HLA-Aw19 and HLA-B5. A two-year prospective study.. Cancer.

[OCR_00527] Sengar D. P., McLeish W. A., Stewart T. H., Harris J. E. (1977). HLA antigens in bronchogenic carcinoma.. Oncology.

[OCR_00538] Weiss G. B., Nawrocki L. B., Daniels J. C. (1980). HLA type and survival in lung cancer.. Cancer.

